# Velocity-selective arterial spin labelling bolus duration measurements: Implications for consensus recommendations

**DOI:** 10.1162/imag_a_00506

**Published:** 2025-03-18

**Authors:** Ian D. Driver, Hannah L. Chandler, Eleonora Patitucci, Emma L. Morgan, Kevin Murphy, Stefano Zappala, Richard G. Wise, Michael Germuska

**Affiliations:** Cardiff University Brain Research Imaging Centre (CUBRIC), School of Psychology, Cardiff University, Cardiff, United Kingdom; Cardiff University Brain Research Imaging Centre (CUBRIC), School of Physics and Astronomy, Cardiff University, Cardiff, United Kingdom; Department of Neurosciences, Imaging and Clinical Sciences, “G. d’Annunzio University” of Chieti-Pescara, Chieti, Italy; Institute for Advanced Biomedical Technologies (ITAB), “G. d’Annunzio University” of Chieti-Pescara, Chieti, Italy; Department of Radiology, University of California Davis Medical Center, Sacramento, CA, United States

**Keywords:** magnetic resonance imaging (MRI), perfusion, cerebral blood flow (CBF), arterial transit time (ATT), Fourier-transform velocity-selective inversion (FT-VSI), cerebrovascular reactivity (CVR)

## Abstract

Velocity-selective arterial spin labelling (VSASL) MRI is insensitive to prolonged arterial transit time. This is an advantage over other arterial spin labelling schemes, where long arterial transit times can lead to bias. Therefore, VSASL can be used with greater confidence to study perfusion in the presence of long arterial transit times, such as in the ageing brain, in vascular pathologies, and cancer, or where arterial transit time changes, such as during measurement of cerebrovascular reactivity (CVR). However, when calculating perfusion (cerebral blood flow, CBF, in the brain) from VSASL signal, it is assumed that a vascular crushing module, defining the duration of the bolus, is applied before the arrival of the trailing edge. The early arrival of the trailing edge of the labelled bolus of blood will cause an underestimation of perfusion. Here, we measure bolus duration in adult, healthy human brains, both at rest and during elevated CBF during CO_2_breathing (5% inspired CO_2_). Grey matter bolus duration was of 2.20 ± 0.35 s/2.22 ± 0.53 s/2.05 ± 0.34 s (2/3/4 cm/s v_cutoff_) at rest, in close agreement with a prior investigation. However, we observed a significant decrease in bolus duration during hypercapnia, and a matched reduction in CVR above a labelling delay of approximately 1.2 s. The reduction in CVR and bolus duration was spatially heterogenous, with shorter hypercapnic bolus durations observed in the frontal lobe (1.31 ± 0.54 s) and temporal lobes (1.36 ± 0.24 s), compared to the occipital lobe (1.50 ± 0.26 s). We place these results in the context of recommendations from a recent consensus paper, which recommends imaging 1.4 s after the label, which could lead to CBF underestimation in conditions with fast flow or during CVR measurements. These results can be used to inform the experimental design of future VSASL studies, to avoid underestimating perfusion by imaging after the arrival of the trailing edge of the labelled bolus.

## 1 Introduction

Arterial spin labelling (ASL) MRI provides a non-invasive measurement of perfusion. It is being used to further our understanding of physiological mechanisms in both the brain (Haller et al., 2016;Liang et al., 2013;Mattsson et al., 2014;Satterthwaite et al., 2014;Segerdahl et al., 2015;Staffaroni et al., 2019) and body (Taso et al., 2023). ASL consists of labelling water protons in arterial blood and waiting for the labelled water to perfuse into the tissue of interest before imaging. The original and predominant ASL method labels water protons based on spatial position, outside of the imaging volume (spatially-selective ASL) (Alsop et al., 2015;Dai et al., 2008;Detre et al., 1992;Edelman et al., 1994;Kim, 1995;Williams et al., 1992;Wong et al., 1998). This approach is dependent on waiting long enough for the labelled blood to flow from the labelled arteries into the tissue of interest, a time defined as the arterial transit time (ATT).

Subsequently, an alternative ASL method was proposed, termed velocity-selective ASL (VSASL), whereby water protons are labelled based on their velocity, rather than their spatial position (Wong et al., 2006). The main advantage of this approach is that blood is labelled much closer to the tissue of interest, significantly reducing or eliminating the ATT (Qin et al., 2022). This insensitivity to ATT makes VSASL a good candidate for studying situations where ATT is long, or may change, which would result in a biased measurement of perfusion for spatially-selective ASL. Long arterial transit times have been observed in the ageing brain (Mahroo et al., 2024;Scheel et al., 2000), in vascular pathologies (Bokkers et al., 2009;Bolar et al., 2019;Fan et al., 2017;Qiu et al., 2012;Wang et al., 2013) and in gliomas (Qu et al., 2022). Changing ATT is also a source of bias in cerebrovascular reactivity (CVR) measurements made with spatially-selective ASL (Zhao et al., 2021). Therefore, there is a large range of applications that could benefit from the ATT insensitivity of VSASL.

VSASL consists of a velocity-selective labelling module followed by a vascular crushing module. The velocity-selective labelling module labels spins moving above a cut-off velocity (v_cutoff_). After a period where the labelled arterial blood spins perfuse into tissue, a vascular crushing module suppresses signal from spins moving above v_cutoff_, leaving the signal from perfused spins which decelerated as they perfused into the tissue. Here, we define the time between the velocity-selective label and the vascular crushing module to be the Label-to-Crusher Time (LCT). The labelling module can either be formed of a train of saturation or inversion pulses. Labelling efficiency is higher with the inversion method and the SNR approaches that of pCASL (Qin et al., 2022). A Fourier transform velocity selective inversion (FT-VSI) labelling method (Qin & van Zijl, 2016) was chosen for this study, with FT-VSI recently demonstrating good test-retest reliability for measuring CBF in healthy participants (Xu et al., 2023). A recent consensus paper recommended LCT = 1.4 s and v_cutoff_= 2 cm/s (Qin et al., 2022).

The bolus duration is the time from when the leading edge of the labelled blood enters the tissue of interest until the trailing edge of the bolus arrives. As no further fresh labelled blood arrives, the kinetic curve decays according to T_1_. Models used to quantify perfusion with a single LCT often assume that the bolus duration is longer than the LCT. If the bolus duration is shorter than the LCT, then these models will overestimate the duration of the labelled bolus and thus underestimate perfusion. Therefore, knowledge of bolus duration will improve specificity of perfusion measurements and will help to inform choice of LCT based on the bolus duration of the population to be studied. Bolus duration has been measured previously (Guo & Wong, 2015) as 2.03 ± 0.08 s using velocity-selective saturation ASL in five young healthy volunteers. However, that study did not report the spatial characteristics of bolus duration or the influence of elevated CBF, as observed in some pathologies with fast flow or during cerebrovascular reactivity (CVR) measurements. A recent study raises questions over bolus duration (Xu et al., 2024), with FT-VSI ASL-based measurements of cerebrovascular reactivity (CVR) being underestimated by 40% when an LCT of 1.52 s was used, but when a shorter LCT of 0.5 s was used CVR was much closer to a phase-contrast based CVR measurement. The observed underestimation in CVR is consistent with the bolus duration being shorter than 1.52 s, especially during hypercapnia, where flow velocity increases. Previous studies generally consider bolus duration to be equivalent to LCT (the time between labelling and vascular crushing modules), implying that the leading edge of the bolus arrives in the tissue instantaneously, or with minimal delay, and that the trailing edge of the labelled bolus is cut off by the vascular crushing module. In this context, LCT and bolus duration are used interchangeably and are often referred to asτ. This assumes that the trailing edge does not reach the tissue of interest before the vascular crushing module. The present study aims to measure bolus duration in healthy participants at rest and during sustained hypercapnia and place these measurements in the context of previous studies and recommended values of LCT.

## 2 Methods

### 2.1 Hardware and general acquisition parameters

Data were acquired on a Siemens 3T Prisma MRI system with body transmit and a 32-channel head receive-only coil. An MPRAGE T_1_-weighted anatomical scan was acquired for image registration and tissue segmentation (TR/TE/TI = 2100/3.19/850 ms; flip angle 8°; 1.1 x 1.1 x 1.0 mm, sagittal orientation (224 x 224 x 176 matrix); phase partial Fourier 7/8).

A Fourier transform velocity selective inversion (FT-VSI) ASL sequence (Qin & van Zijl, 2016) was implemented with regional pre-sat (2 s pre-delay) using the water suppression enhanced through the*T*_1_effects (WET) technique (Ogg et al., 1994), and a sym-BIR-8-based vascular crushing module. v_cutoff_was defined as perQin et al. (2022)and values of 2, 3 and 4 cm/s were used in*Experiment 1*, while 3 cm/s was used in*Experiment 2*. The FT-VSI pulse train was 62 ms in length with composite refocusing pulses, velocity-compensated control, and a 7.8 ms gap between each 20° hard pulse; the rise/fall time for the gradients in the FT-VSI pulse train was 0.35 ms. The gradient amplitude was altered linearly to achieve the desired v_cutoff_, with an amplitude of 18.75 mT/m used to achieve a 2 cm/s v_cutoff_in the inferior-superior direction.

Three background suppression pulses were timed to ensure the perfusion contrast was proportional to the magnitude subtraction between tag and control conditions at all LCTs, avoiding the need for complex image subtraction. The timings of the background suppression pulses were interpolated from the results presented inLiu et al. (2020), and inversion times are given inTable 1.

**Table 1.** Background suppression timings, delay from the end of the labelling module to the centre of the inversion pulse.

LCT (ms)	Inversion 1 (ms)	Inversion 2 (ms)	Inversion 3 (ms)
550	288	323	353
660	329	426	476
800	380	557	632
960	439	706	808
1150	508	833	1016
1390	596	1107	1275
1670	699	1368	1574
2000	820	1677	1919
2400	967	2050	2329
2900	1150	2517	2828
3500	1370	3077	3407

LCT is the time from the labelling module to the vascular crushing module.

A 2D EPI readout was used, with 3.3 x 3.3 mm in-plane resolution (64 x 64 matrix), 15 slices (6 mm thickness, 3 mm slice gap), TE = 12 ms, and 6/8 partial Fourier. TR was set as short as possible, ranging from 3.5–6.3 s from the shortest to longest LCT. A 50 ms delay was applied between the vascular crushing module and the readout to reduce eddy currents, and the post-label delay increased by 42.5 ms for each ascending slice. Four tag/control pairs were acquired for each LCT with an M_0_acquired at the start of each set. LCT values will be specified in each respective experimental section below.

The M_0_included the sym-BIR-8-based vascular crushing module to cancel out T_2_weighting in the perfusion quantification. The first M_0_in each block was preceded by a pause of more than 30 s, so it was free from T_1_weighting and was used for quantification.

### 2.2 Experiment 1: Bolus duration measurements

Thirteen healthy participants (8 female, 5 male; aged 18–52, median 22, mean ± stdev 28 ± 11 years) gave written informed consent. The School of Psychology, Cardiff University Ethics Committee approved this study.

To calculate bolus duration, 11 LCT values of [0.55, 0.66, 0.8, 0.96, 1.15, 1.39, 1.67, 2, 2.4, 2.9, 3.5] s were acquired in a total time of 7:20. This was repeated for three v_cutoff_values of 2/3/4 cm/s, with the v_cutoff_order balanced across participants (as there were 13 participants, 5 participants had an order 2/3/4, whereas 4 participants each underwent orders of 3/4/2 and 4/2/3 cm/s).

### 2.3 Experiment 2: Bolus duration response to hypercapnia CVR

Nineteen age-matched healthy participants (10 female, 9 male; aged 18–45, median 22, mean ± stdev 26 ± 9 years) gave written informed consent. The School of Psychology, Cardiff University Ethics Committee approved this study. The sample size for Experiment 2 was powered a-priori based on the mean grey matter bolus duration measurements from Experiment 1 and anticipating a reduction in bolus duration of 0.1 s with hypercapnia (based on a similar reduction in bolus arrival time measured in pCASL and PASL previously) (Donahue et al., 2014;Ho et al., 2011;Su et al., 2017). For an effect size of 0.7, alpha = 0.05, and power = 0.8, a total sample size of 19 was required (G*Power 3.1).

The gas delivery system used was introduced previously by our group (Germuska et al., 2016), with mass flow controllers (MKS Instruments, Wilmington, MA, USA) delivering either 25 L/min of medical air (normocapnia) or 5% CO_2_(hypercapnia). Participants wore a close-fitting face mask (Quadralite, Intersurgical, Wokingham, Berkshire, UK) attached to a breathing circuit following the design of Tancredi and colleagues (Tancredi et al., 2014). Exhaled gas was monitored throughout, with a sampling line from the face mask to a rapidly responding gas analyzer (PowerLab, ADInstruments, Sydney, Australia).

To calculate bolus duration, a set of eight LCT values of [0.55, 0.66, 0.8, 0.96, 1.15, 1.39, 1.67, 2] s were acquired in a total time of 4:45. This set was repeated four times, [normocapnia – hypercapnia – normocapnia – hypercapnia]. To increase participant compliance, the amount of time a participant was at hypercapnia in each set was minimised by omitting the last three LCT values (2.4, 2.9, and 3.5 s) acquired in*Experiment 1*. Each set only started once end-tidal PCO_2_stabilised to a steady state (~1 min following the transition), such that each condition lasted approximately 6 min. An M_0_image was acquired at the start of each set, more than a minute after the preceding acquisition, so it was free from T_1_weighting. A 3 cm/s v_cutoff_was chosen to reduce cerebrospinal fluid (CSF) signal contamination, which is a potential problem for hypercapnic acquisitions, where global vasodilation displaces CSF volume (Blockley et al., 2011;Thomas et al., 2013).

### 2.4 Data analysis

FT-VSI ASL data were concatenated across LCT values for each dataset, then motion correction was performed (MCFLIRT, FSL (Jenkinson et al., 2002)). Bolus duration and CBF were estimated using a two-compartment perfusion model adapted from Buxton et al. (1998) (‘general kinetic model’), assuming instantaneous arrival of the bolus in the tissue (Equation 1). An additional macrovascular component, as perChappell et al. (2010), was included to account for any remaining signal in the arterial or venous vessels that is not completely removed by the BIR-8 vascular crushing module (Equation 2). Model fitting was performed in MATLAB via unconstrained minimisation (Quasi-Newton method). A multi-start implementation (with multiple starting values for bolus duration and macrovascular bolus duration) was used to reduce the likelihood of fitting to a local minimum.

Equation 1

$$\begin{array}{l} \Delta M(t)=2\cdot \alpha \cdot \alpha_{BGS}\cdot f\cdot \tau \cdot M_{o}\cdot e^{\frac{-t}{T_{1,blood}}}\cdot q_{p}(t)\\ q_{p}(t)=\frac{e^{kt}(1-e^{-k\tau })}{k\tau }\\ k=\frac{1}{T_{1,blood}}-\frac{1}{T_{1}^{'}}\;\;\;\;\;\;\;\;\;\;\;\;\;\;\;\;\;\;\frac{1}{T_{1}^{'}}=\frac{1}{T_{1}}+\frac{f}{\lambda } \end{array}$$

τ= bolus duration whenτ≤LCT, otherwiseτ=LCT

Equation 2

$$\begin{array}{cc} \Delta M_{macro}(t)=2\cdot \alpha \cdot \alpha_{BGS}\cdot macroBV\cdot M_{0}e^{\frac{-t}{T_{1,blood}}} & t\leq \tau_{macro}\\ \Delta M_{macro}(t)=0\;\;\;\;\;\;\;\;\;\;\;\;\;\;\;\;\;\;\;\;\;\;\;\;\;\;\;\;\;\;\;\;\;\;\;\;\;\;\;\;\;\;\;\;\;\;\;\;\;\;\;\;\;\;\;\;\;\;\;\;\;\;\;\; & \tau_{macro}<t \end{array}$$

Where ∆M is the signal difference between the label and control images.τ= LCT for the case where LCT≤bolus duration. However,τ= bolus duration in the case where LCT > bolus duration (i.e., the image is acquired after arrival of the trailing edge of the labelled bolus).$\tau_{macro}$is the macrovascular bolus duration, and macroBV is the apparent macrovascular blood volume fraction (blood volume signal remaining after application of the vascular crushing module). t is the time between labelling pulse and readout (i.e., LCT + post-label delay for each slice), f is the cerebral blood flow (ml/g/s), α is the inversion efficiency (0.56), α_BGS_is the additional signal attenuation caused by the three background suppression pulses (0.86),λis the blood-tissue-partition coefficient (0.9 ml/g), M_0_is the signal intensity of the proton density image, T_1,blood_= 1.6 s is the T_1_value of blood (Lu et al., 2004), and tissue T_1_= 1.3 s (Wansapura et al., 1999).

M_0_scans were coregistered to the MPRAGE using FSL epi_reg (Greve & Fischl, 2009;Jenkinson et al., 2002), and the MPRAGE was coregistered to MNI152 space (Fonov et al., 2011) using FSL FNIRT (Jenkinson et al., 2012).

Grey matter maps were segmented using FSL BET (Smith, 2002) and FAST (Zhang et al., 2001) on the MPRAGE. Grey matter partial volume estimate maps were realigned into native ASL space. All grey matter analyses were performed using a mask of voxels with a grey matter partial volume estimate of at least 50%. An additional threshold was applied to the M_0_of greater than 50% of the maximum signal to avoid areas where the EPI signal had significant signal drop-off (intra-voxel dephasing close to air spaces).

The MNI Structural Atlas (Mazziotta et al., 2001), which is provided in FSL in MNI152 space, was realigned into native ASL space. Regional analyses were performed within the grey matter mask defined in the previous paragraph by defining each region based on a 25% threshold of that region’s partial volume estimate (Craddock et al., 2012). All region analyses were calculated by taking the median across voxels. Summary data were reported as mean and standard deviation across participants.

Group average bolus duration and CBF maps were calculated in MNI152 space by realigning parameter maps into MNI152 space at 2 mm isotropic resolution, then taking the mean across participants. For physiological consistency, estimates of bolus duration and macrovascular bolus duration were assigned a value of zero when the respective CBF or macrovascular blood volume estimates were zero or less.

## 3 Results

In Experiment 1, results are first presented in the context of how bolus duration can inform the choice of sequence timings (LCT, the labelling to vascular crushing delay time) at rest, presenting data from all three v_cutoff_values in parallel. Secondly, the effects of small changes in v_cutoff_are considered in the context of designing CVR experiments. Finally, results are presented from Experiment 2, examining the impact of hypercapnia on the choice of sequence timings for CVR experiments or in populations with fast flow.

### 3.1 Experiment 1a: Bolus duration measurements

**Fig. 1.**
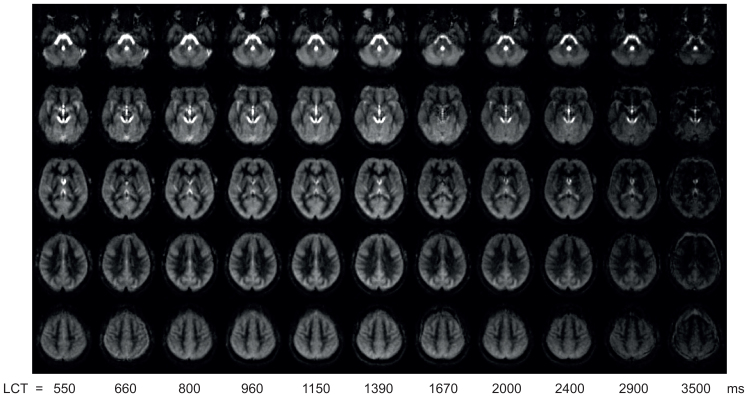
∆M maps from same 5 slices in group space for each LCT acquired at rest in Experiment 1. The five slices shown are at MNI152 z = -32, -12, 8, 28, 48 mm.

Figure 1shows group-averaged maps of ∆M in MNI space for each LCT acquired with a v_cutoff_of 2 cm/s (data for 3 and 4 cm/s can be found inSupplementary Figure S1).

**Fig. 2.**
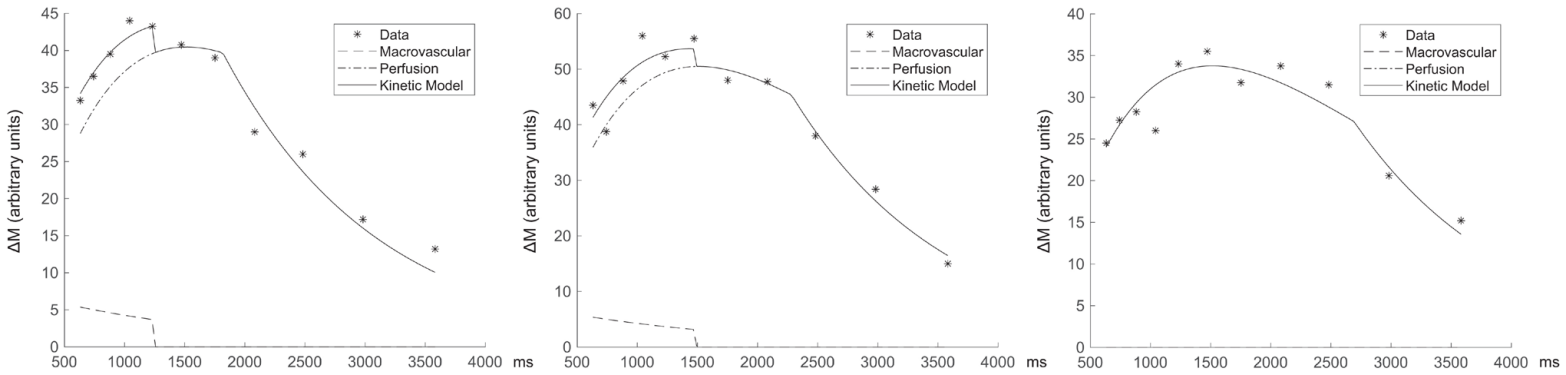
Example kinetic model fits to mean grey matter signal for three volunteers. Chosen examples have short (1.73 s), medium (2.20 s), and long (2.61 s) bolus durations. Each figure displays the raw data and the kinetic model fits, including the perfusion signal, macrovascular signal, and combined kinetic model. The first two examples show evidence of partial voluming with a macrovascular signal with a shorter macrovascular bolus duration.

Fitting the kinetic model ofEquations 1and2to the average grey matter signal reveals a bolus duration that is shorter than the maximum LCT.Figure 2shows example model fits for three subjects, demonstrating short (1.73 s), medium (2.20 s), and long (2.61 s) bolus durations. In many subjects, there is evidence of partial voluming with a macrovascular blood signal with a shorter macrovascular bolus duration, suggesting incomplete suppression of macrovascular blood by the BIR-8 crushing module. There was no discernible evidence of a bolus arrival time in grey matter, consistent with the idea that the leading edge of the velocity selective bolus is created close to the tissue. However, we were unable to verify this observation in white matter due to the significantly reduced SNR.

Averaging voxelwise fits across grey matter and participants provides bolus duration estimates (mean ± standard deviation across participants) of 2.20 ± 0.35 s/2.22 ± 0.53 s/2.05 ± 0.34 s at rest (2/3/4 cm/s v_cutoff_). Grey matter CBF values were 57 ± 8 ml/100 g/min/56 ± 8 ml/100 g/min/54 ± 9 ml/100 g/min (2/3/4 cm/s v_cutoff_). The observed macrovascular blood volume was, on average, less than 0.2% with a macrovascular bolus duration of 1.22 ± 0.17 s/1.22 ± 0.11 s/1.21 ± 0.13 s (2/3/4 cm/s v_cutoff_). All participants’ grey matter CBF, bolus duration, macrovascular blood volume, and macrovascular bolus duration, with median and interquartile range are shown inFigure 3. Where macrovascular blood volume was zero, it was not possible to characterise macrovascular bolus duration, so these voxels were not included in grey matter averages.

**Fig. 3.**
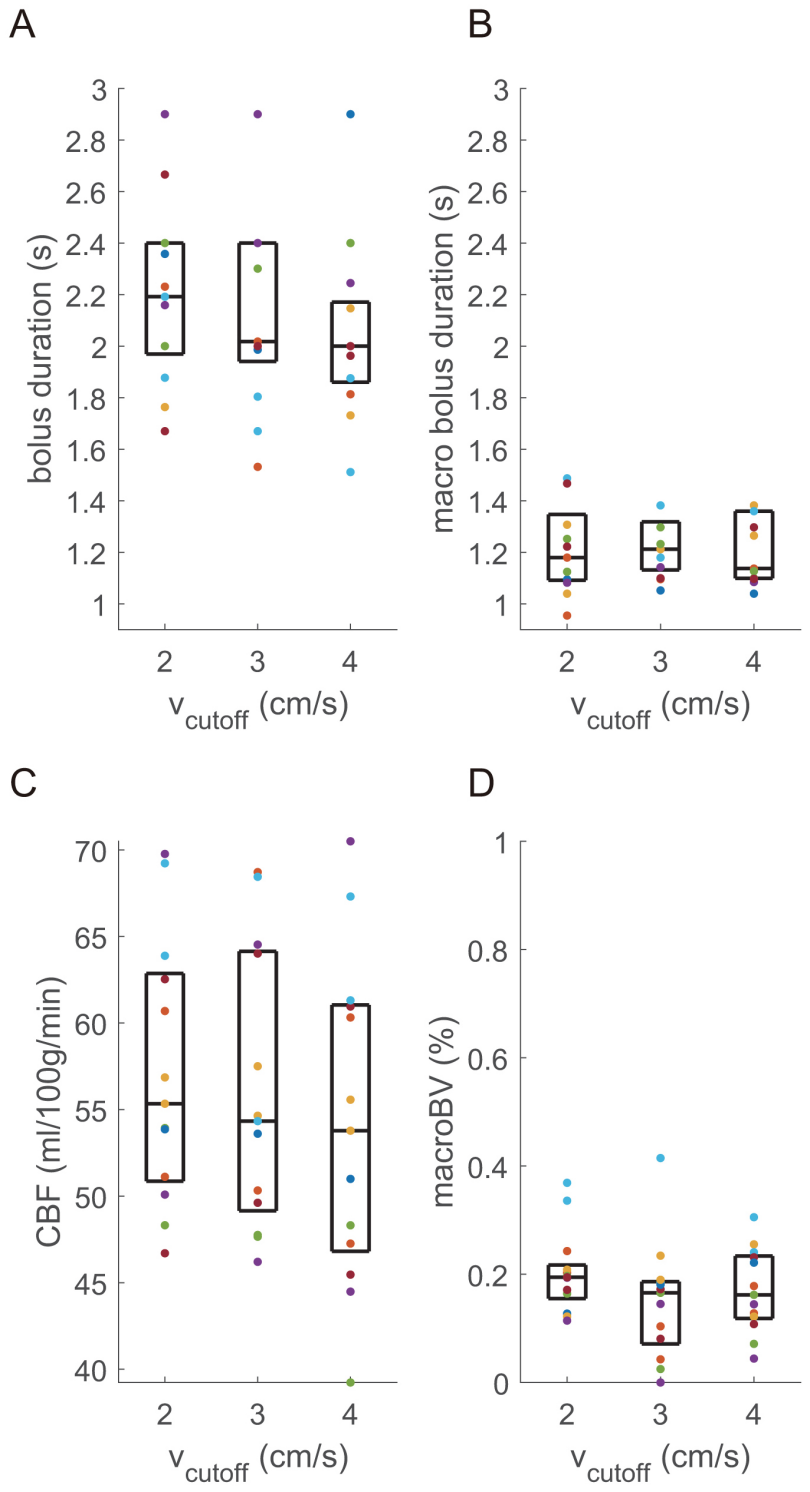
(A) Grey matter bolus duration [s] for each v_cutoff_(box shows median and interquartile range, points show each participant’s data). (B) Grey matter macrovascular bolus duration [s]. (C) Grey matter CBF [ml/100 g/min]. (D) Grey matter macrovascular blood volume [%].

**Fig. 4.**
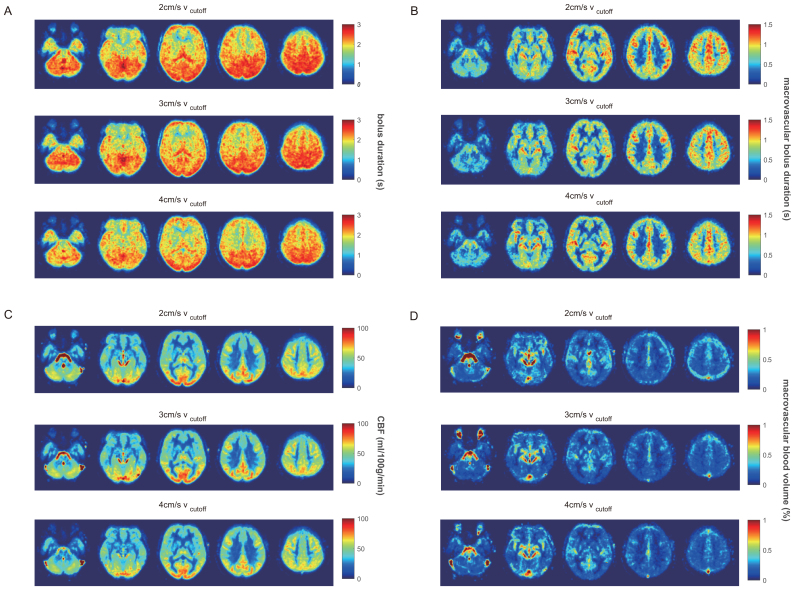
(A) Group bolus duration [s] maps for each v_cutoff_(B) Mean macrovascular bolus duration [s]. (C) Group mean CBF maps [ml/100 g/min]. (D) Group mean macrovascular blood volume maps [%]. The five slices shown are at MNI152 z = -32, -12, 8, 28, 48 mm.

Bolus duration maps inFigure 4Ashow spatial heterogeneity, with anterior and temporal regions having shorter bolus duration than posterior regions. The low white matter macrovascular bolus duration values inFigure 4Bare an artefact of being unable to characterise macrovascular bolus duration in voxels with zero macrovascular blood volume. Macrovascular bolus duration was set to zero for voxels with zero macrovascular blood volume, before transformation to group space and averaging across participants. Maps of macrovascular blood volume (Fig. 4D) are mostly consistent with regions with known macrovascular anatomy. However, in contrast to PASL experiments (Chappell et al., 2010), the maps also show some venous contrast, which is likely associated with venous outflow during the 2D readout.

The spatial heterogeneity is further investigated by subdividing the brain into 9 MNI regions (Mazziotta et al., 2001), including the four brain lobes, cerebellum, and four subcortical structures. The shortest bolus duration was found in the caudate, 1.58 ± 0.24 s/1.60 ± 0.34 s/1.77 ± 0.51 (2/3/4 cm/s v_cutoff_), while the longest bolus duration was observed in the thalamus, 2.35 ± 0.59 s/2.75 ± 0.76 s/2.24 ± 0.57 (2/3/4 cm/s v_cutoff_).Table 2summarises the regional parameter estimates for both Experiments 1 and 2 (which is discussed in detail inSection 3.3). All regions in Experiment 1 have a bolus duration longer than the consensus recommended LCT = 1.4 s (Qin et al., 2022), suggesting that the consensus recommendation is, indeed, appropriate for the cohort investigated at rest (normocapnia).

**Table 2.** Summary of group parameter estimates (mean value ± standard deviation across participants) for Experiments 1 and 2, divided into MNI lobes and subcortical structures.

	Bolus duration (s)					CBF (ml/100 g/min)					
	Experiment 1			Experiment 2		Experiment 1			Experiment 2		
Region	2 cm/s	3 cm/s	4 cm/s	Air	CO _2_	2 cm/s	3 cm/s	4 cm/s	Air	CO _2_	CVR (%/mmHg)
Caudate	1.58 ± 0.24	1.60 ± 0.34	1.77 ± 0.51	1.41 ± 0.31	1.15 ± 0.30	35 ± 9	36 ± 8	32 ± 6	36 ± 9	47 ± 14	6.4 ± 13.1
Cerebellum	2.41 ± 0.46	2.33 ± 0.51	2.16 ± 0.51	1.86 ± 0.17	1.54 ± 0.26	53 ± 9	50 ± 9	47 ± 10	50 ± 10	77 ± 17	8.0 ± 7.0
Frontal lobe	2.02 ± 0.26	1.94 ± 0.30	1.90 ± 0.38	1.56 ± 0.24	1.31 ± 0.25	52 ± 8	52 ± 8	52 ± 10	56 ± 13	82 ± 22	7.1 ± 7.5
Insula	1.88 ± 0.49	1.89 ± 0.59	1.95 ± 0.45	1.42 ± 0.25	1.25 ± 0.26	43 ± 4	42 ± 6	39 ± 7	48 ± 7	63 ± 18	5.2 ± 7.4
Occipital lobe	2.39 ± 0.48	2.30 ± 0.56	2.14 ± 0.26	1.76 ± 0.20	1.50 ± 0.26	71 ± 11	71 ± 11	68 ± 12	67 ± 15	96 ± 22	6.8 ± 6.3
Parietal lobe	2.38 ± 0.42	2.31 ± 0.52	2.15 ± 0.40	1.68 ± 0.20	1.46 ± 0.25	66 ± 7	65 ± 9	63 ± 11	67 ± 14	95 ± 20	6.6 ± 6.3
Putamen	1.92 ± 0.76	2.03 ± 0.74	1.94 ± 0.80	1.44 ± 0.28	1.25 ± 0.32	37 ± 11	36 ± 11	29 ± 11	40 ± 12	52 ± 16	6.0 ± 6.3
Temporal lobe	2.05 ± 0.44	2.07 ± 0.53	2.01 ± 0.51	1.56 ± 0.23	1.36 ± 0.24	53 ± 8	52 ± 7	50 ± 8	54 ± 11	72 ± 14	5.4 ± 5.8
Thalamus	2.65 ± 0.59	2.75 ± 0.76	2.24 ± 0.57	1.76 ± 0.21	1.49 ± 0.27	63 ± 17	58 ± 14	55 ± 12	53 ± 15	88 ± 23	10.2 ± 7.8
Grey matter	2.20 ± 0.35	2.22 ± 0.53	2.05 ± 0.34	1.65 ± 0.20	1.43 ± 0.24	57 ± 8	56 ± 8	54 ± 9	58 ± 11	83 ± 17	6.7 ± 6.2

### 3.2 Experiment 1b: Impact of v _cutoff_ for designing CVR experiments

Reviewing the above results in the context of minimising signal contamination from CSF spaces,Figure 1andSupplementary Figure S1demonstrate that the apparent perfusion signal from CSF spaces is reduced from 2 cm/s to 3 cm/s v_cutoff_and reduced to a lesser extent from 3 cm/s to 4 cm/s v_cutoff_. WhileFigures 3–4suggest that if there are any differences in CBF and bolus duration measured at each v_cutoff_, then they are small compared to intra- and inter-participant variation. A one-way ANOVA testing for a change in grey matter bolus duration across v_cutoff_was not significant, F(2,36) = 0.72; p = 0.50. Therefore, for CVR experiments, where CSF spaces are apparently displaced by vasodilation, a higher v_cutoff_of 3 cm/s was chosen. This is higher than the consensus recommendation 2 cm/s (Qin et al., 2022) but is likely to be more appropriate in this context.

### 3.3 Experiment 2: Bolus duration response to hypercapnia CVR

**Fig. 5.**
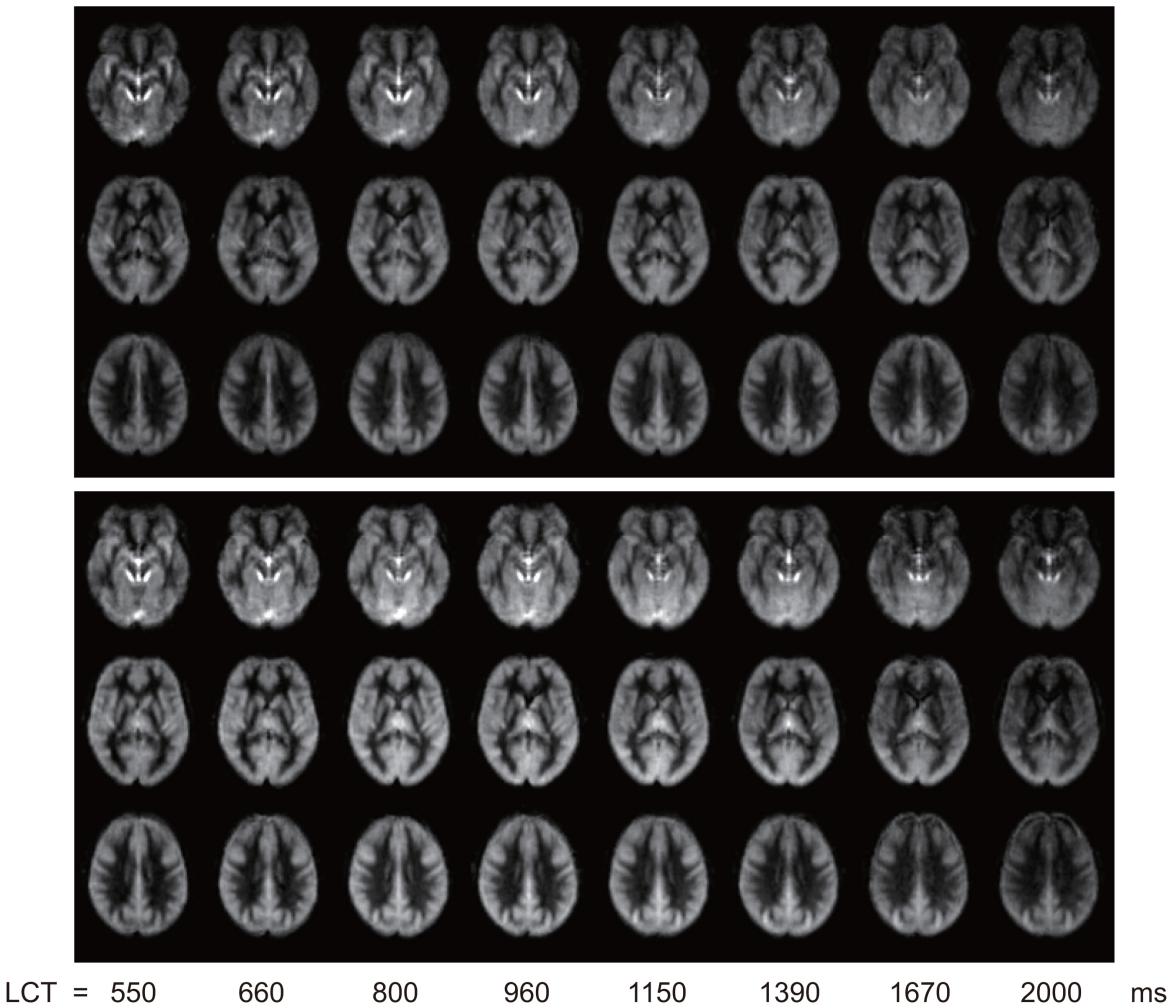
∆M maps from same 3 slices in group space for each LCT acquired at rest in Experiment 2. Top panel is data acquired at rest and the bottom panel is that acquired during hypercapnia. The three slices shown are at MNI152 z = -12, 8, 28 mm.

The hypercapnic challenge caused an 8.3 ± 2.4 mmHg increase in P_ET_CO_2_.Figure 5shows group-averaged maps of ∆M in MNI space for each LCT acquired at rest and during hypercapnia.

**Fig. 6.**
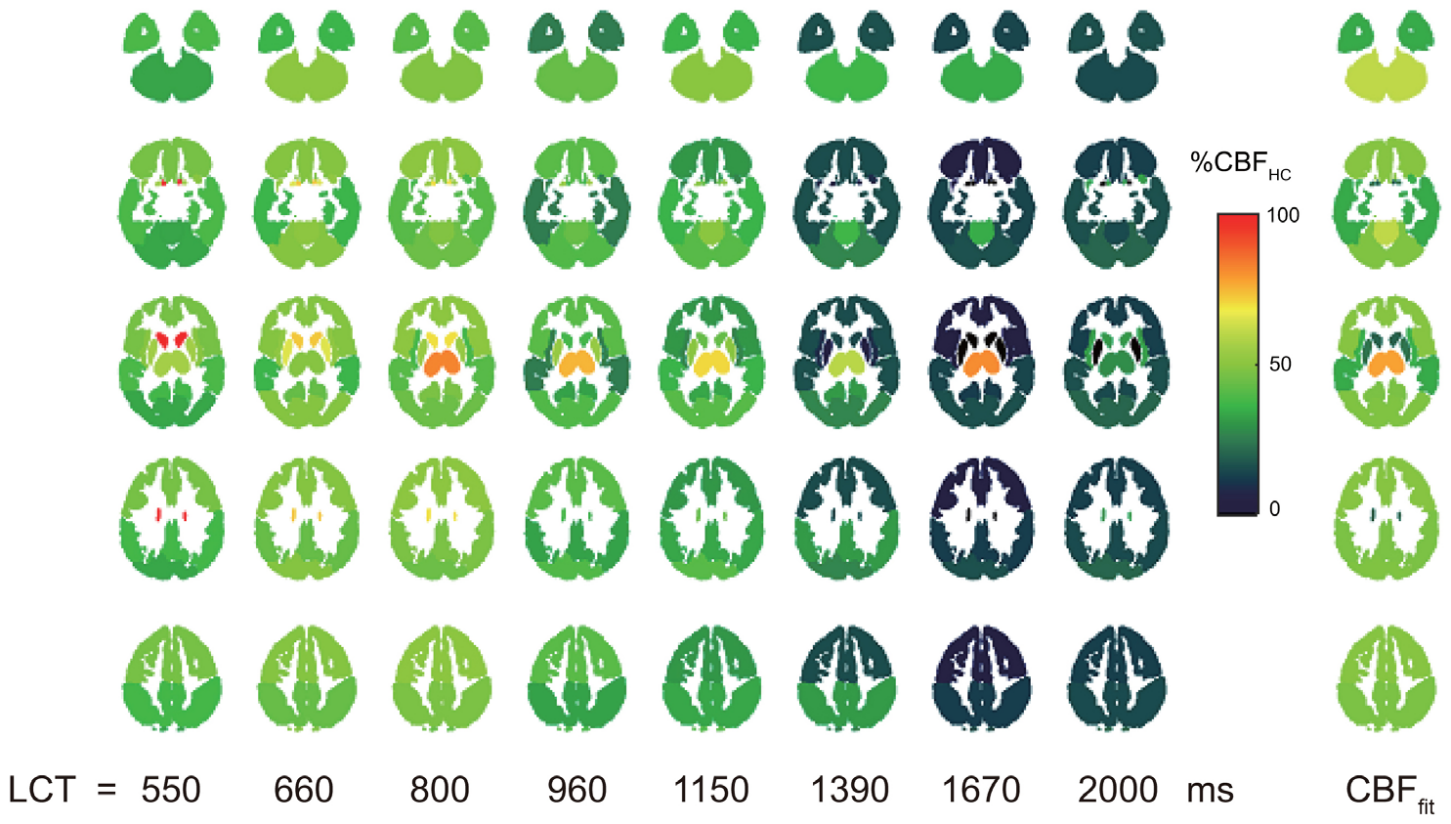
Median percentage increase in ∆M, averaged across MNI lobes and subjects for each LCT. For comparison, the CBF increase calculated with the kinetic model (CBF_fit_) is displayed. CBF_fit_and the shorter LCTs ∆M maps show increased signal change during hypercapnia compared to ∆M maps acquired with LCT greater than approximately 1150 ms.

Fitting the data to the kinetic model (Equations 1and2) produced an estimated grey matter CBF increase of 45 ± 23% during hypercapnia and a CVR of 6.7 ± 6.2%/mmHg (seeTable 2).Figure 6shows that the median increase in perfusion signal with hypercapnia is reduced for longer LCTs. Visual inspection of the CVR maps suggests a large underestimation of CVR when using an LCT above approximately 1.2 s. GM CVR across LCTs was 6.8 ± 11.9, 6.2 ± 5.2, 6.7 ± 6.0, 6.8 ± 11.3, 6.9 ± 10.4, 5.0 ± 7.3, 2.8 ± 5.5, and 3.4 ± 5.8%/mmHg for LCTs 550/660/800/960/1150/1390/1670/2000 ms. Thus, we find that CVR is reduced by approximately 25.4%, 58.7%, and 50.0% for LCTs of 1390 ms/1670 ms/2000 ms compared to LCTs from 550 to 1150 ms.

The grey matter bolus duration measured in Experiment 2 was shorter both at rest (normocapnia) 1.65 ± 0.20 s and during hypercapnia 1.43 ± 0.24 s (seeTable 2). The shorter bolus duration at rest is undoubtedly a result of the limited sampling of longer LCTs during Experiment 2 (a requirement of the experiment to maintain subject comfort and compliance during prolonged hypercapnia). Truncating the data in Experiment 1 (Vcutoff = 3 cm/s) at a maximum LCT of 2.0 s reduces the mean GM bolus duration estimate from 2.22 ± 0.53 s to 1.69 ± 0.12 s. However, the mean CBF_0_estimate is not significantly affected with values of 56 ± 8 ml/100 g/min and 61 ± 9 ml/100 g/min. The histograms inSupplementary Figure S2demonstrate how bolus duration estimates are truncated at 2.0 s (the longest LCT) in Experiment 2 but not Experiment 1, with many estimates hitting the 2.0 s limit during normocapnia. Therefore, sampling of LCTs was not sufficient to quantify the bolus duration accurately. However, during hypercapnia the increased blood flow velocity appears to reduce the bolus duration significantly, with far fewer estimates hitting the 2.0 s limit. Indeed, with the modal peak appearing around 1.4 s, it appears that the sampling of LCTs is sufficient to capture the hemodynamics during hypercapnia, with only a small negative bias expected from group-averaged results.

**Fig. 7.**
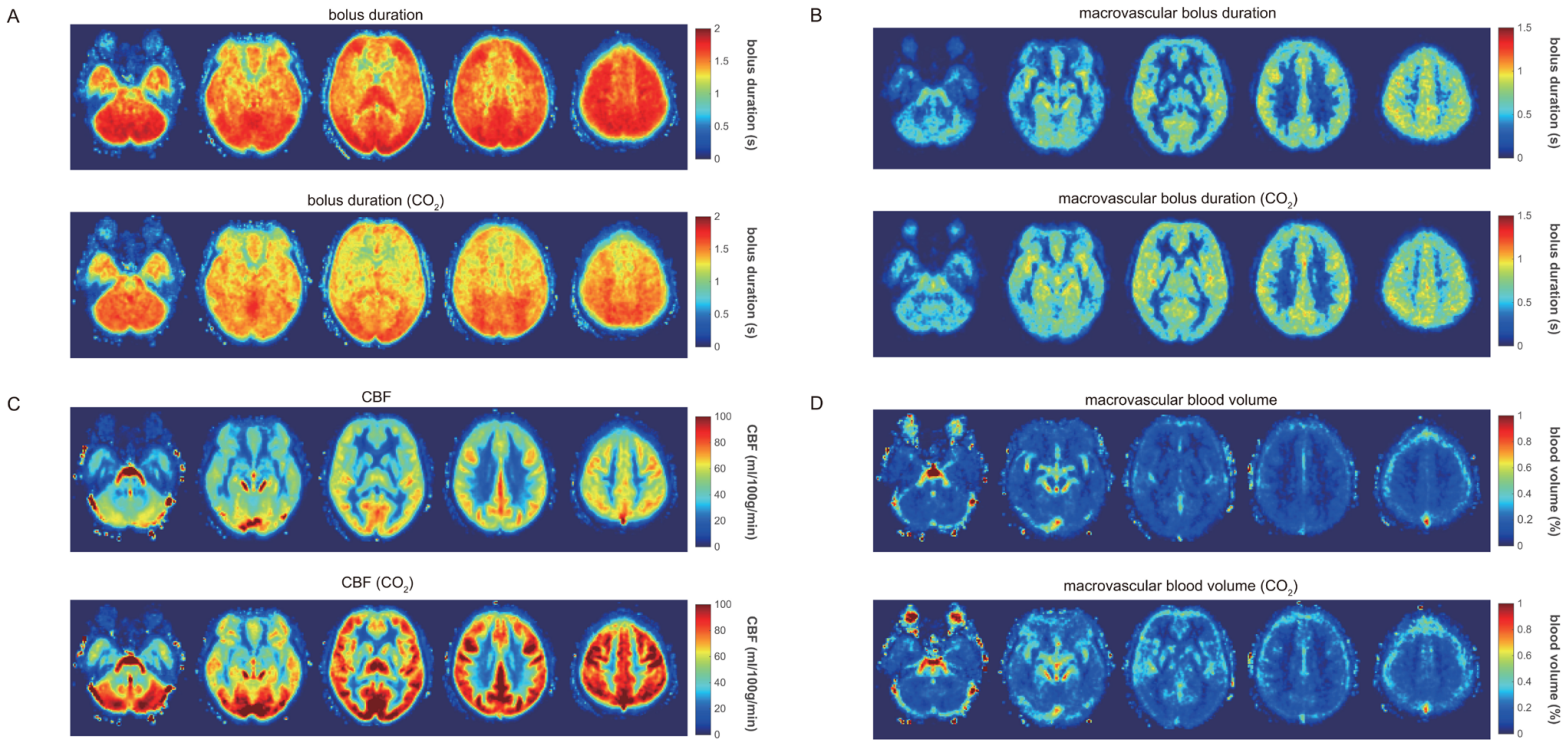
Top row—parameter maps during air breathing (averaged across all participants). Bottom row—parameter maps during 5% CO_2_hypercapnia for (A) bolus duration (note the reduced colour scale compared toFig. 2); (B) macrovascular bolus duration; (C) CBF; (D) macrovascular blood volume. The five slices shown are at MNI152 z = -32, -12, 8, 28, and 48 mm.

Figure 7shows the group-averaged maps of the kinetic model fits, demonstrating the significant reduction in bolus duration during hypercapnia, with shorter bolus duration in the frontal and temporal regions compared to posterior regions during hypercapnia. The average bolus duration map during normocapnia is more uniform due to the 2.0 s upper LCT limit. There is no observable change in the macrovascular bolus duration between normocapnia and rest, while there appears to be an increase in the macrovascular blood volume, with GM macrovascular blood volume being 0.10 ± 0.07 at normocapnia and 0.15 ± 0.07% at hypercapnia (mean ± std over participants).

Expanding the regional analysis to the MNI lobes (Table 2), we can see that the minimum average bolus duration is 1.15 ± 0.30 s in the caudate, with many regions including both the frontal and temporal lobes having a bolus duration less than 1.4 s (the consensus recommended LCT). Therefore, consistent with the results shown inFigure 6, the bolus duration measurements suggest that an LCT of less than 1.4 s is needed to accurately capture CVR. Indeed, an upper limit of approximately 1.2 s appears to be appropriate in this adult cohort.

## 4 Discussion

This work presents measurements of bolus duration in the healthy adult brain for Fourier-transform velocity selective inversion ASL. We show that while grey matter average bolus duration is 2.2 s at rest, there is a large spatial variability, with anterior and temporal regions having shorter bolus durations than posterior regions. We extend these findings by measuring a reduction in bolus duration during hypercapnia in an experiment designed to measure CVR, providing an example where high flow velocity causes a reduction in bolus duration. These results help to understand the origin of reported underestimation of CVR with FT-VSI ASL when using conventional LCTs (Xu et al., 2024), and enable an upper bound to be placed on LCT for such experiments.

The motivation for longer LCT is that more labelled blood can perfuse into tissue, increasing signal. Therefore, the optimal LCT is a balance of specificity (shorter LCT) and sensitivity (longer LCT). Future VSASL studies can take these results to inform the choice of LCT based on bolus duration for their population and region of interest. For example, in the healthy, predominantly female cohort studied in*Experiment 1*, the consensus recommendation LCT of 1.4 s appears appropriate for all anatomical regions at rest and is expected to provide unbiased CBF estimates across the brain. However, for CVR experiments an LCT of 1.2 s (or less) appears to be more appropriate, being the lower limit of the bolus duration measured during hypercapnia, as it would provide an unbiased measurement of CBF change across all cortical and sub-cortical regions. In cohorts where fast flow velocity can occur during normocapnia, for example, anaemia (Gottesman et al., 2012), subarachnoid haemorrhage (Uryga et al., 2022), and traumatic brain injury (Kelly et al., 1996), then it is likely that a shorter LCT will also be required to avoid CBF underestimation.

Both at rest and during hypercapnia a clear pattern of reduced bolus duration in frontal and temporal regions was observed and is consistent with shorter ATT in the anterior circulation, compared to the posterior circulation, that has been observed using pCASL (Pinto et al., 2023;Zhao et al., 2021). This pattern of reduced regional bolus duration is also evident in the results ofXu et al. (2024), where the largest CVR reduction was observed in these regions with a LCT of 1.52 s. The reduction in GM bolus duration during*Experiment 2*, (CVR acquisition) was approximately 13%, which is comparable with ATT reductions recently reported with pCASL during hypercapnia (Pinto et al., 2023). However, this is likely to be an underestimate as bolus duration measurements are limited to 2.0 s in our hypercapnia experiments, which is insufficient for characterising the bolus duration at rest.

Secondary to measuring bolus duration,*Experiment 1*also investigated the effect of v_cutoff_on the FT-VSI ASL signal. Minimizing v_cutoff_brings the label into smaller arteries and arterioles, closer to the capillary bed. This extends the leading edge of the labelled bolus and minimises the contribution of small blood vessels to the subtracted ASL signal. However, signal contamination from CSF is present with low v_cutoff_values. Thus, the choice of v_cutoff_is somewhat a trade-off between CSF contamination and the contribution of small blood vessels to the subtracted ASL signal. While there appears to be a small, non-significant reduction in bolus duration with v_cutoff_changing from 2 to 4 cm/s (Fig. 3), this is small compared to intra- and inter-participant variability in bolus duration, while CBF measurement was not affected by v_cutoff_. However, increasing v_cutoff_above 2 to 3 cm/s resulted in reduced CSF signal contamination with a smaller reduction apparent moving from 3 to 4 cm/s (Supplementary Fig. S1). Therefore, considering the trade-off between CSF contamination and the desire to minimise blood vessel contribution, we chose to use a v_cutoff_of 3 cm/s for*Experiment 2*, where CSF contamination could confound measurement of CBF CVR, where CSF spaces are displaced by global vasodilation (Blockley et al., 2011;Thomas et al., 2013). In the case where a patient group may have atrophy, or for CVR studies (Zhao et al., 2021), our results support using a v_cutoff_of 3 cm/s to reduce CSF contamination, while not significantly reducing the size of the labelled bolus.

While visible in the lateral ventricles, CSF also borders cortical regions, so it will form a small fraction of many of the voxels classified as grey matter, contributing to the signal of these voxels. Therefore, a consideration of the nature of the CSF signal is important to understand if CSF signal contamination could bias the bolus duration calculation in cortical regions. The CSF signal has large variability due to its sensitivity to physiological noise, especially cardiac pulsations. This high variability, combined with multiparametric fitting to the limited number of LCT datapoints, meant that we were unable to fit a CSF component in the multi-compartment model. However, the T_1_of CSF is long compared to the perfusion kinetics, so CSF signal is relatively constant across the range of LCT values acquired here, as demonstrated inFigure 1. The contribution of physiological noise in the CSF signal adds a large degree of variation across measurements, but there is no dependence of this variability on LCT.

A limitation of our method for measuring bolus duration is that we assume a sharp trailing edge of the labelled bolus which does not account for any dispersion. Dispersion would smooth out the kinetic curve. The effect of dispersion in the data, not accounted for in the model kinetic curve, would be to overestimate bolus duration. Additionally, we chose to fit a macrovascular compartment to minimise any bias from residual macrovascular signal that was not completely supressed due to the macrovascular crushing gradients being applied in only one direction (foot-head). It is possible that this component is not a pure macrovascular signal but also contains some contribution from diffusion attenuation effects that are not completely matched between the velocity-selective label and control. The effect of any diffusion contribution to this compartment would be to introduce error into the macrovascular bolus duration. However, it is unlikely that such errors would have a significant influence on the principal parameters of interest, CBF and bolus duration.

A further consideration is the use of slab-selective pre-saturation, rather than global pre-saturation. Slab-selective pre-saturation has the potential benefit of increased SNR for CVR ASL experiments, where we wish to minimise the TR so that the acquisition is sensitive to CBF changes over a short time scale. The SNR benefit is due to the assumption that all arterial blood is fresh blood and, therefore, has fully relaxed longitudinal magnetisation, whereas the magnetisation is reduced by a factor of 1 – exp(-Tsat/T_1,blood_) when using global pre-saturation (Tsat = saturation time). A regional pre-saturation scheme was also used in the work ofXu et al. (2024), and further investigation is needed to determine if this has an influence on the bolus duration under conditions of fast flow velocity.

In conclusion, bolus duration in FT-VSI reduces significantly with increased flow velocity, as demonstrated with a hypercapnic intervention in a cohort of healthy adults. Thus, the use of a shorter LCT will be important when studying groups or states where bolus duration is short or expected to change, such as cerebrovascular reactivity mapping, anaemia, subarachnoid haemorrhage, and traumatic brain injury. Our data suggest that an LCT of 1.2 s or less will minimise any bias in CVR measurements in healthy adult populations, although at the expense of reduced signal-to-noise in superior and posterior brain regions that exhibit a longer bolus duration. We also found that increasing v_cutoff_from 2 to 3 cm/s did not have a significant effect on either bolus duration or CBF quantification. However, there is a clear reduction in CSF contamination, which, if present, could bias cerebrovascular reactivity measurements, or confound CBF quantification in certain pathologies. Therefore, we recommend increasing FT-VSI v_cutoff_to at least 3 cm/s for such studies.

## Supplementary Material

Supplementary Figure 1

Supplementary Figure 2

## Data Availability

Participants only gave ethical approval for anonymised data to be shared, so we are unable make the raw ASL or MPRAGE data publicly available. Instead, we provide bolus duration and CBF parameter maps for each participant, in group (MNI152) space, and summary grey matter and Harvard-Oxford atlas region-averaged data, available athttps://doi.org/10.17605/OSF.IO/6SJNQ. The analysis code for estimating CBF and bolus duration is available onlinehttps://doi.org/10.5281/zenodo.13755864.
