# Supplementary material for: Velocity-selective arterial spin labelling bolus duration measurements: Implications for consensus recommendations
Source: Imaging Neurosci (Camb). 2025 Mar 18;3:imag_a_00506. doi: 10.1162/imag_a_00506 (PMC7617564; doi:10.1162/imag_a_00506)
Supplement: Supplementary Figure 1 [file imag_a_00506-supp1.pdf]

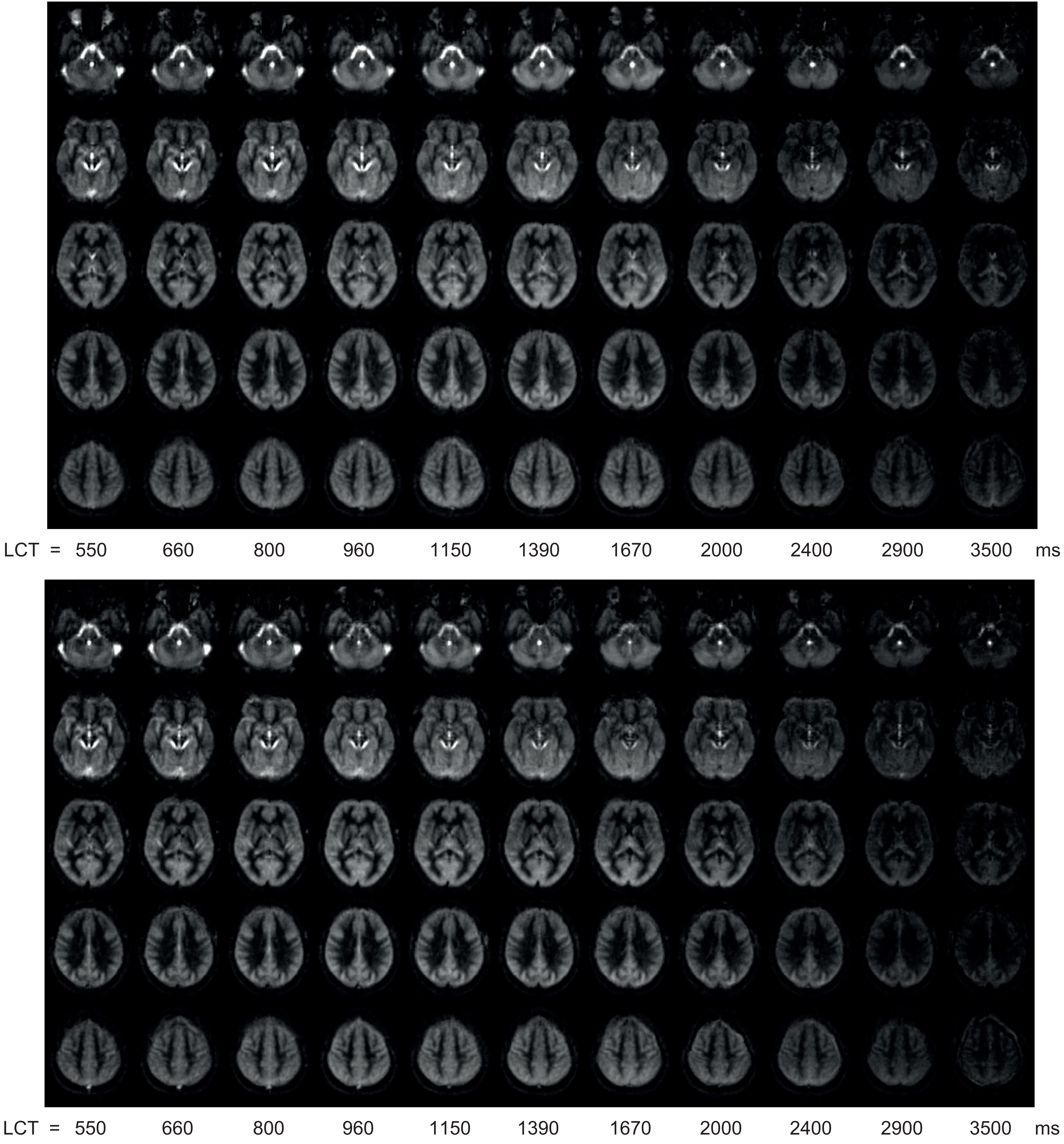

Figure S1. Top panel Vcut = 3 cm/s, bottom panel Vcut = 4 cm/s. ΔM maps from same 5 slices in group space for each LCT acquired at rest in experiment 1. The five slices shown are at MNI152 z = -32, -12, 8, 28, 48 mm
