# Supplementary material for: Velocity-selective arterial spin labelling bolus duration measurements: Implications for consensus recommendations
Source: Imaging Neurosci (Camb). 2025 Mar 18;3:imag_a_00506. doi: 10.1162/imag_a_00506 (PMC7617564; doi:10.1162/imag_a_00506)
Supplement: Supplementary Figure 2 [file imag_a_00506-supp2.pdf]

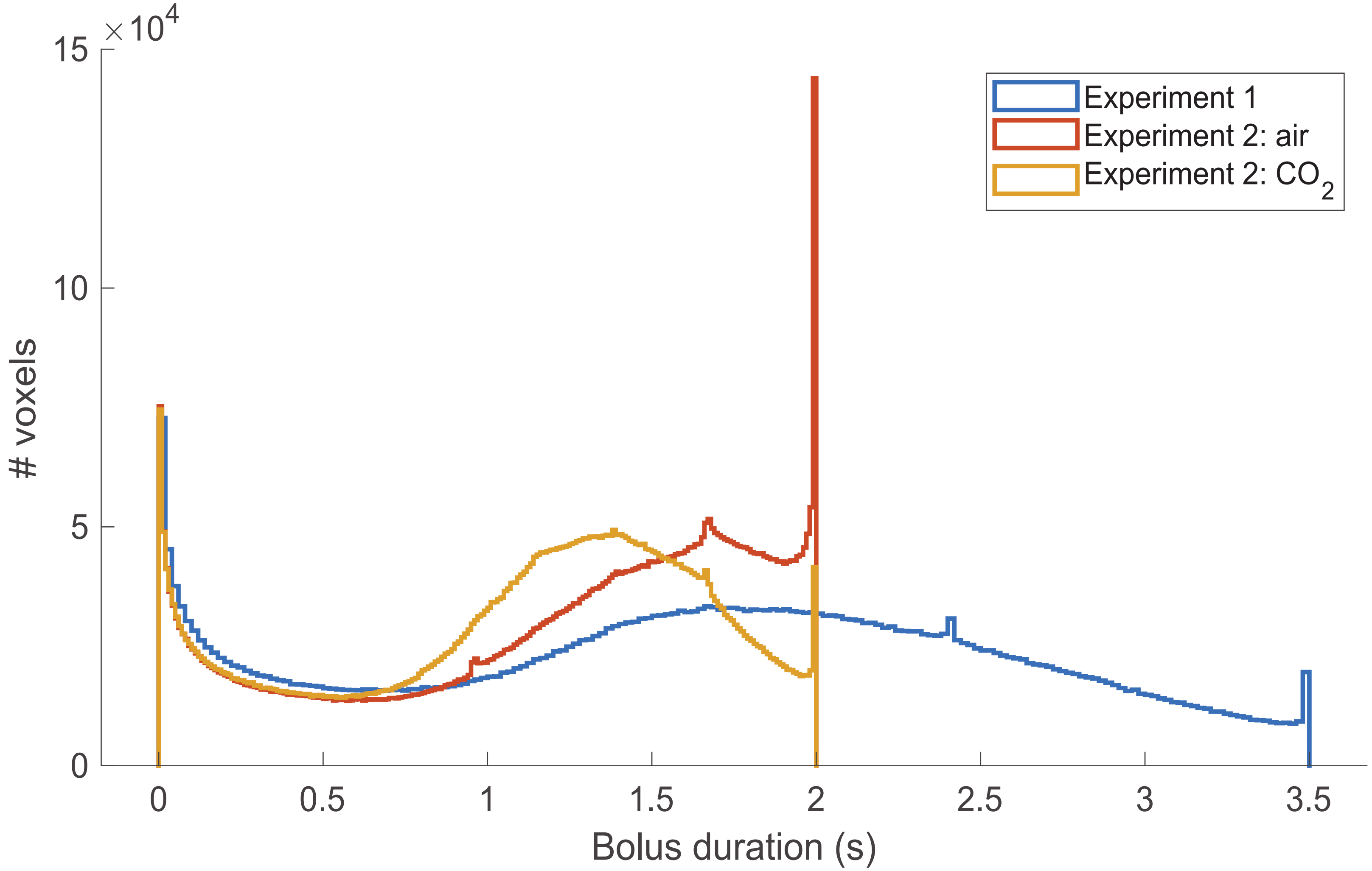

Figure S2. Histogram of bolus duration estimates for all subjects. The distribution of bolus duration estimates is limited to the maximum LCT acquired, 3.5 s for experiment 1 and 2.0 s for experiment 2. The 2.0 s limit is too low to capture the full range of bolus duration estimates during normocapnia in experiment 2, with many estimates hitting the 2.0s limit. During hypercapnia most of the bolus duration estimate fall below the 2 s limit and the modal value is effectively captured by the limited LCT sampling.
